# Influence of Internal Architecture and Ink Formulation on the Thermal Behavior of 3D-Printed Cementitious Materials

**DOI:** 10.3390/ma17235736

**Published:** 2024-11-23

**Authors:** Michael Kosson, Lesa Brown, Garrett Thorne, Florence Sanchez

**Affiliations:** 1 Department of Chemical and Biomolecular Engineering, Vanderbilt University, PMB 351604, 2301 Vanderbilt Place, Nashville, TN 37235-1604, USA; michael.t.kosson@vanderbilt.edu; 2 Department of Civil and Environmental Engineering, Vanderbilt University, PMB 351831, 2301 Vanderbilt Place, Nashville, TN 37235-1831, USA; lesa.brown@vanderbilt.edu; 3 The Wond’ry, Vanderbilt’s Innovation Center, Vanderbilt University, 2414 Highland Ave Suite 102, Nashville, TN 37212-2010, USA; garrett.thorne@vanderbilt.edu

**Keywords:** extrusion 3D printing, cement paste, halloysite nanoclay, thermal properties, internal architecture

## Abstract

Cement-based 3D printing provides an opportunity to create cement-based elements with a hierarchy of structures and patterns that are not easily achievable using traditional casting techniques, thereby providing new possibilities for improving thermal control and energy storage in cement-based materials. In this study, the influence of internal architecture and ink formulation on the thermal behavior of 3D-printed cement composite beams was investigated using infrared thermal imaging and a conceptual one-dimensional heat transfer model based on cooling fins in convective media. Three-dimensional printed beams with rectilinear, three-dimensional honeycomb, and Archimedean chord infill patterns and cement ink formulations with and without 5% halloysite nanoclay were exposed to a heating source at one end. The thermal behavior of the beams was found to be predominantly influenced by their internal architecture rather than the cement ink formulation, with differences in void structures and heat transfer pathways among the different architectures resulting in a hierarchy of apparent thermal conductivity. The internal architecture resulted in a reduction in apparent thermal conductivity by up to 75%, while the incorporation of halloysite nanoclay in the cement ink led to a reduction of up to 14%. Among the tested internal architecture, the rectilinear architecture showed a 10–15% higher apparent thermal conductivity compared to the three-dimensional honeycomb architecture and a 35–40% higher apparent thermal conductivity than the Archimedean architecture. The research demonstrates a promising strategy for fabricating and evaluating cement-based materials with thermal management capabilities using 3D printing methods.

## 1. Introduction

As the global energy demand for heating and cooling in buildings continues to increase and is projected to account for almost half of the total growth in energy demand by 2050, thermal energy performance of building envelopes has drawn increased attention. The development of new materials and use of innovative construction methods that facilitate thermal control and energy storage have been identified as particularly important to reduce the energy demand of buildings and enable a shift to decarbonized buildings [1]. In this context, there has been considerable interest in optimizing the thermal properties of cement-based materials (paste, mortar, and concrete) due to their low cost, mechanical strength, and versatility as construction materials. Recent research has focused on enhancing the thermal properties of cement-based materials by modifying their microstructure. This can be achieved by altering the pore structure and/or by adding inclusions that can absorb changes in thermal conditions, such as phase-change materials and thermal insulation materials. Various methods have been employed to adjust the thermal conductivity of cement-based materials by modifying the pore structure [2]. These methods include the use of air-entraining additives, lightweight and porous aggregates, and chemically active additives such as bentonite nanoclay, silica fume, or fly ash. Air-entraining additives, for example, introduce microscopic air bubbles within the cement matrix, effectively reducing the material’s density and thermal conductivity, enhancing insulation properties [3,4,5]. Lightweight and porous aggregates, such as expanded clay, pumice, and perlite, are also used to replace traditional dense aggregates, further lowering the thermal conductivity and improving the material’s thermal performance [4,6,7,8,9,10,11,12,13]. Additionally, chemically active additives such as bentonite nanoclay, silica fume, and fly ash are utilized to refine the microstructure of cement-based composites. Bentonite nanoclay, with its high surface area and unique layered structure, creates a tortuous path for heat flow, which reduces heat transfer and enhances the insulation properties, thereby improving the material’s overall thermal performance [14]. Silica fume and fly ash are used to fill pores and act as pozzolanic materials, reacting with calcium hydroxide to form calcium silicate hydrates [15,16,17,18]. Together, these actions contribute to a denser and more refined microstructure, which can both enhance thermal resistance and mitigate potential adverse effects on mechanical strength that often accompany thermal optimization. Phase-change materials (PCMs), such as paraffin wax and fatty acids (organic) and salt hydrates and metallic materials (inorganic) [19], have also been utilized in cement-based applications. PCMs are substances that can absorb and release thermal energy during the process of melting and freezing, making them effective for regulating temperature. When incorporated into cement-based materials, PCMs can enhance energy efficiency by storing excess heat during periods of high temperatures and releasing it when temperatures decrease. However, while the integration of PCMs offers these benefits, it can alter the microstructure, potentially insufficiently dispersing or creating voids that compromise the material integrity, leading to a reduction in mechanical strength and durability. Additional challenges include thermal stability and compatibility with the cement matrix [20]. The advantages and disadvantages of existing methods for controlling the thermal properties of cement-based materials, along with the need to reduce their environmental impact, provide opportunities for the development of novel technologies in this area.

Recent advances in additive manufacturing and 3D printing of cement-based materials have opened up new possibilities for the fabrication of novel, architected cement-based materials that can have a hierarchy of structures and patterns otherwise not achievable with traditional casting techniques. Three-dimensional printing may provide a new avenue for enhancing the thermal energy performance of building envelopes and develop new thermal energy storage systems by enabling the design of cement-based materials with local control of the material’s internal architecture, including shape, size, connectivity, and local porosities. There are a variety of infill patterns available for 3D printing, including hexagonal, concentric, rectilinear, and Archimedean chords, which are used to print “solid” builds at various infill percentages. By designing how the material is organized and deposited in successive layers during printing, different types of structural and thermal performance can be achieved. Three-dimensional printing provides a unique opportunity to tune the material’s thermal anisotropy and directionality of the heat flow within the material’s internal structure and can therefore help develop cement-based elements for improved thermal control and energy storage. Despite several studies that have used computational modeling to investigate the relationship between internal geometry (type of infill patterns and cross-sections) and thermal performance [21,22,23,24,25,26,27], generally demonstrating a strong relationship, experimental investigations on 3D-printed cement-based materials have, however, remained limited [28,29,30,31], and more study to quantify the relationship experimentally is needed. In the meantime, the incorporation of nanoclays in 3D printing cement inks has shown great potential in controlling the rheology and enhancing the hardened properties of printed cement-based materials [32,33,34,35,36,37,38,39,40,41,42,43,44]. While some insulating properties have also been shown, comparatively little attention has been paid to the effect of nanoclay incorporation on the thermal properties of cement-based materials. Furthermore, most available studies have reported on the effect of nanoclays at temperatures that are higher (up to 1000 °C) than those relevant for building envelope efficiency [14,45,46,47]. Among the different types of nanoclays, halloysite nanoclay (HNC), a two-layered aluminosilicate with a predominantly hollow nanotubular structure (i.e., needle-like structure), is of particular interest as it may provide internal reinforcement through nanocrack bridging [48,49].

The novelty of this research resides in the experimental characterization of the effect of internal architectures and HNC incorporation on the thermal behavior of 3D-printed cement composites. HNC was chosen for this study because prior research by the authors has demonstrated its improvements to both printability and hardened mechanical properties in cement-based 3D printing [50]. Additionally, its high aspect ratio was considered for its potential to induce preferential alignment during the printing process. This alignment is an interesting avenue for optimizing the mechanical and thermal performance of the printed structures, as it contributes to anisotropic behavior and can allow for tunable thermal characteristics. A 5% dosage was chosen for this study, despite being potentially higher than the optimal level for enhancing the cement ink mechanical performance, in order to effectively assess its impact on thermal performance.

The internal architecture of the 3D-printed material can affect its thermal behavior by influencing its ability to conduct heat. Moreover, the thermal behavior of the material can be changed by adjusting the cement ink formulation. Architected cement beams with a wall thickness of one filament (approximately 1.6 mm) and three internal architectures—rectilinear (RL), three-dimensional honeycomb (3DHC), and Archimedean chords (AC)—were 3D-printed with cement ink formulations with and without 5% cement replacement with HNC. Solid cast beams were also fabricated as references for comparison. The thermal behavior of the 3D-printed architected cement beams and solid cast beams was characterized by integrating thermal experiments and real-time infrared thermal imaging with the transient and steady-state analytical solutions of a conceptual one-dimensional heat transfer fin model specifically adapted for this study. This innovative approach allowed the complexities of the internal architectures to be considered while maintaining an analytical form that facilitates a direct comparison of the relative effective thermal conductivity across different beam internal architectures and ink formulations. Consequently, the presented methodology enables practical and accessible exploration of diverse design possibilities and provides a simple yet effective tool for evaluating thermal performance in material and structural design.

## 2. Materials and Methods

### 2.1. Materials

Type I/II portland cement (OPC) from LafargeHolcim, Chicago, IL, USA was employed as the binding agent for the cement inks. To ensure printability, the following three additives were incorporated: MasterGlenium 7700 (BASF, Cleveland, OH, USA), a superplasticizer that improves workability for filament formation; MasterMatrix VMA 362 (BASF, Cleveland, OH, USA), a viscosity-modifying admixture; and MasterMatrix UW 450 (BASF, Cleveland, OH, USA), used to prevent paste segregation during extrusion. Halloysite nanoclay (HNC) (Sigma-Aldrich, St. Louis, MO, USA), was used to control the rheology and setting time of the cement ink, as well as to affect its thermal capacity. HNC—chemical composition Al_2_Si_2_O_5_(OH)_4_—is a material comprising bi-layer sheets with internal aluminol and external siloxane surfaces, rolled to form a needle-like structure measuring 30–70 nm in diameter and 1–3 µm in length. It has a surface area of 64 m^2^/g and 1.3 mL/g porosity.

### 2.2. Architected Cement Composite Preparation

#### 2.2.1. Cement Ink Design

Two types of cement inks were utilized: a “neat” cement paste ink (OPC ink) and an ink that had 5% of the OPC replaced with HNC (HNC ink) (Table 1). These ink designs provided adequate ink qualities for consistent printability in preliminary testing and were within the manufacturer’s recommended dosages for admixtures [51,52,53]. The admixtures were added to the water and sonicated using a 500 W probe sonicator (Fisher Scientific Model 505 Sonic Dismembrator, Hampton, NH, USA) for 20 s at 50% power amplitude. This procedure was found to be sufficient to effectively disperse the admixtures. When applicable, HNC powder was added to the OPC powder and stirred until visibly homogenous. Then, the HNC/OPC powder combination was mixed for 1 min at 400 RPM with a stainless steel paddle bit in a mounted corded drill with rotation speed controlled by a DC motor. The homogenized water/admixture mix was added to the HNC/OPC powder and mixed for 1 min with rotation speed ramping up from 400 RPM to 1000 RPM, followed by 2 min at 1000 RPM. This mixing procedure was found to consistently result in well-mixed, visually homogeneous cement inks.

#### 2.2.2. Three-Dimensional Printed and Cast Cement Beam Fabrication

**Figure 1:**
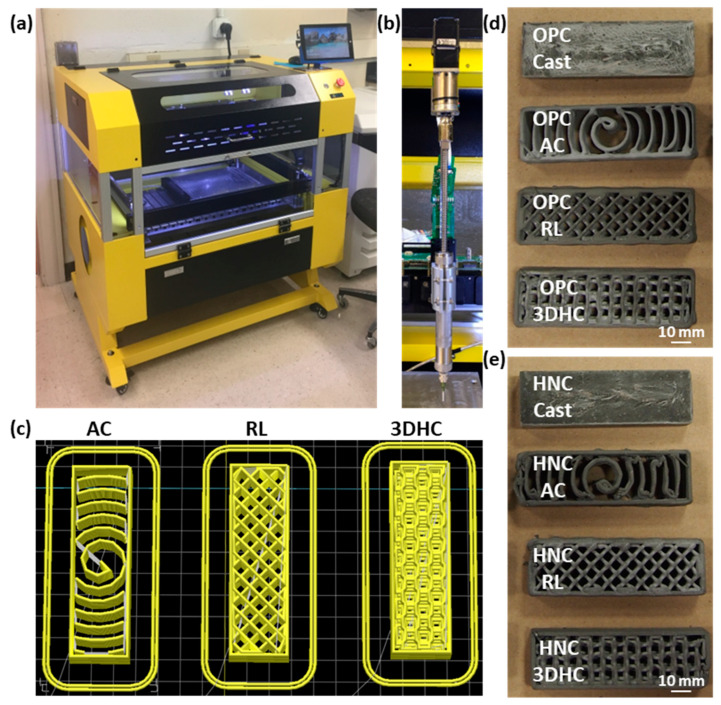
(**a**) Gantry-style 3D printer; (**b**) extrusion-based printing head assembly; (**c**) toolpath schematics of the infill patterns utilized for 3D-printed beams to create internal architecture: Archimedean chords (AC), rectilinear (RL), and three-dimensional honeycomb (3DHC); (**d**) cast and 3D-printed specimens made with the OPC ink; and (**e**) cast and 3D-printed specimens made with the HNC ink.

All 3D-printed cement beams were printed using a Hydra 430 gantry model 3D printer with Repetrel 3D printing software, version 42.400, and EMO-XT modular 3D print heads (Hyrel 3D, Norcross, GA, USA; Figure 1a,b). A .STL file for a beam measuring 90 mm long, 28 mm wide, and 14 mm high was generated, and slicing recipes with one vertical perimeter layer, no horizontal perimeter layers, a layer height of one mm, and three infill patterns were produced using Slic3r slicing software (version 1.3.0, Slicer, Italy). This beam size was selected to maximize the use of the cement ink that could be loaded into the print heads. The following three infill patterns with 30% infill and a variety of filament pathways and void structures for heat transfer were selected: rectilinear (RL)—such that each successive layer was printed in an orthogonal alignment from the previous layer; three-dimensional honeycomb (3DHC)—such that the extrusion produced interlocking hexagonal voids resulting in a planar section of alternating small squares and octagons; and Archimedean chords (AC)—such that the extrusion pattern spiraled outward from the center of the sample (Figure 1c). Cavity size varied depending on infill pattern (despite a constant infill percentage), but in all cases, filaments within the same layer were separated by approximately 7 mm. This separation affected thermal conductivity by limiting conductive exchanges and changing the profile of convective and radiative exchanges.

The OPC cement ink was manually loaded into the 3D print head. A vibrating table was used to aid loading with the HNC ink, which was more viscous than the OPC ink. Tamping with a 1.6 mm diameter stainless steel rod mitigated air bubbles. Samples were 3D-printed 14 layers high, which yielded a beam-shaped sample that was approximately 90 mm long, 28 mm wide, and 14 mm high. Three replicates were printed for each internal architecture and cement ink, totaling eighteen 3D-printed samples.

As a control, each cement ink was also cast into beam-shaped silicon molds and tamped with a 1.6 mm stainless steel rod to produce solid beams measuring 90 mm long, 28 mm wide, and 14 mm high. Three replicates of each cement composite type were cast. All beams were aged for 35 days in a curing chamber before characterization.

### 2.3. Thermal Measurements

**Figure 2:**
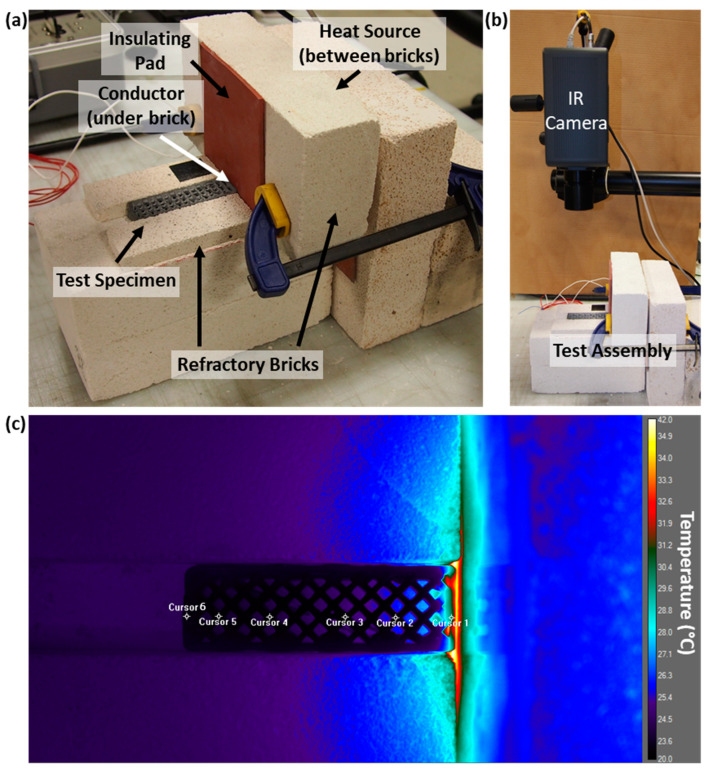
(**a**) Refractory brick sample holder; (**b**) heat transfer experiment set-up; and (**c**) infrared image showing selection of six locations near the centerline for temperature monitoring on a 3DHC beam at time t = 0.

To quantify the effects of ink composition and internal architecture on thermal behavior, an experimental setup was designed where a thermal conductor made of aluminum in contact with a heating element at 60 °C was applied to one end of a test beam, which was either 3D-printed or cast. Three replicates for each architecture and ink type were evaluated using this setup. Refractory bricks were utilized to insulate the bottom and sides of the beam, reducing the effect of heat flow through the beam walls and making the flow of heat through the system more effectively one-dimensional. This setup was designed to direct heat more consistently from the heating element into the beam, while allowing for some heat dissipation from the top surface, in line with the assumptions of the analytical heat transfer model introduced in Section 2.4 (Figure 2a). A FLIR A8300sc MWIR high-performance infrared camera was mounted above the sample to track temperature progression during testing (Figure 2b). This camera, along with its processing software (ResearchIR, version 4.40.2.10, Teledyne FLIR, CA, USA), allowed for quantitative monitoring of surface temperature. An insulating rubber pad was placed above the exposed portion of the conductor to mitigate the effect of the conductor on IR images. The conductor was allowed to preheat to steady state before testing, with the surface to be exposed to the test sample registering between 40.0 and 40.5 °C with the infrared camera. Test samples were coarsely sanded on the sides to reduce printing heterogeneity and improve contact with the heating element and refractory blocks. The samples were allowed to equilibrate to room temperature overnight before testing. They were then exposed to conductive heating for 120 min while being imaged by the infrared camera. The entire test setup was enclosed with acrylic sheets to minimize the effect of room temperature fluctuations and air flow, such as those caused by cycling air conditioning in the laboratory. This experimental setup enabled the quantitative measurement of heat transfer through the material, thereby allowing for a systematic comparison of different combinations of internal architectures and cement ink formulations in terms of their thermal behavior. Thermal images were collected at 10 s intervals for the duration of testing. Image resolution was set to 1130 × 490 pixels. Cursors were placed in six locations along the beam length near the centerline (Figure 2c) for temperature monitoring using the infrared camera software, with average temperature for a nine-pixel square recorded at 10 s intervals. These locations were selected such that they could be at nearly the same distance from the heat source across all beams and be positioned in the center of a printed filament for all patterns. There was some variation due to the differences in available locations between architectures, but they were placed approximately equidistantly, with the first cursor in the middle of the filament in contact with the heat source, and the last cursor on the opposite wall. Distances from the edge of the sample were approximated for the cast beams to be equivalent to the 3D-printed beams.

### 2.4. Analytical Model for Heat Transfer in the 3D-Printed and Cast Cement Beams

**Figure 3:**
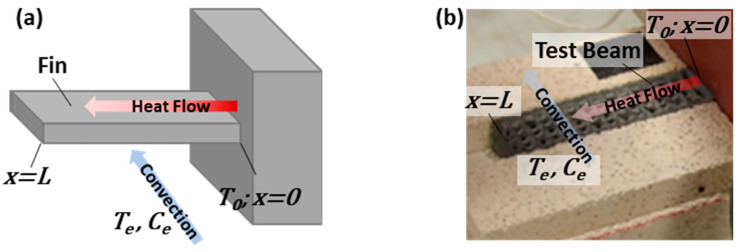
(**a**) Schematic of a fin with a length of *L* that extends from a heated wall with temperature *T*_0_ into a convective medium with temperature *T_e_* and velocity *C_e_*. The temperature of the fin is a function of the distance from the heat source (*x*). This schematic is shown to be conceptually similar to (**b**), the test beam setup.

For modeling purposes, the beams were conceptualized as one-dimensional cooling fins, with one end of the fin attached to a heated wall and the remaining portion exposed to a convective cooling environment, as shown in Figure 3. This model closely resembles the experimental setup, where the beam was in contact with a heating source and exposed to the air environment on its top surface. In this configuration, heat conduction primarily occurs along the length of the fin, while the temperature changes across the width and height of the fin are relatively small compared to the temperature change along its length. This simplifies the analysis to one-dimensional heat transfer. Temperature profiles were monitored at points positioned in the middle of the width of the beam. Although this model has limitations in capturing the complexities associated with convective and radiative heat transfer for the 3D-printed blocks that are characterized by air cavities, it enables an overall characterization of the heat transfer and an apparent thermal conductivity, consolidating various factors into a single coefficient. As discussed below, several parameters were simultaneously fitted by the model, increasing the degrees of freedom to account for these complexities, while still providing a reproducible evaluation of the thermal properties of the beams, allowing for direct comparison. By using the temperature profiles obtained experimentally for each cursor to initialize the unsteady-state model, the overall impact of the internal architecture and ink composition on heat flow could be evaluated without the need to directly model the air cavities or filament shapes, as well as the filament microstructure, which is too complex to fully characterize. Initially, the temperature changes over time at different cursor locations along the beams were modeled in unsteady-state conditions to approximate the steady-state temperature at each point. Next, the approximated steady-state temperatures were utilized to model the temperature profiles of the beams in steady-state conditions as a function of the distance from the heat source. These temperature profiles were subsequently used to determine the apparent thermal conductivity for each beam. This “apparent thermal conductivity” is a lumped parameter that incorporates conduction, convection, and radiative heat transfer into a single coefficient for simplified analysis. This approach allows for a comprehensive assessment of overall heat transfer characteristics within the 3D-printed structures and provides a convenient way to express their thermal performance without breaking down individual contributions from each heat transfer mode. Utilizing this approach facilitates a direct comparison of the relative thermal performance among various beam internal architectures and ink formulations.

The simplified version of the analytical solution of transient heat transfer in a cooling fin provided by [54] was used. This solution meets Fourier’s equation, with the initial condition that the fin is in thermal equilibrium with its surroundings. This solution employs Green’s functions, which involve an infinite summation series. For simplicity, the boundary condition case where the end of the beam is insulated was used. This approximation was appropriate since the temperature change at the end of the beam was small compared to that closer to the heat source. This suggests that the temperature at the end of the beam was not significantly higher than the surrounding environment compared to closer to the heating element, providing little driving force for heat to flow out through the end of the beam, with most heat escaping through the exposed top surface. Consequently, the temperature in the beam as a function of distance from the heat source and time can be determined as follows [54]:

(1) $$T\left(x,\;t\right)-T_{e}=\frac{q_{0}L}{k}\frac{\left(e^{-m\left(2L-x\right)}+e^{-mx}\right)}{mL\left(1-e^{-2mL}\right)}-\frac{q_{0}L}{k}\frac{e^{-m^{2}\alpha t}}{m^{2}L^{2}}\;\;-2\frac{q_{0}L}{k}\sum_{n=1}^{\infty }\frac{cos(\beta_{n}x/L)}{m^{2}L^{2}+\beta_{n}^{2}}\cdot [\exp\left[-\frac{\left(m^{2}L^{2}+\beta_{n}^{2}\right)\alpha t}{L^{2}}\right]],$$

where *T*(*x,t*) is the fin temperature as a function of distance from the heat source *x* and time *t*; *T_e_* is the convective environment temperature; *q*_0_ is the rate of heat flow into the fin; *L* is the length of the fin; *k* is the conductive heat transfer coefficient; *α* is a time constant; and *m* is a dimensionless fin constant where $m\equiv \sqrt{\frac{hA_{h}}{kV}}$ with *h* [MLT^−3^K^−1^] being the convective heat transfer coefficient, *A_h_* [L^2^] the surface area for convection, *V* [L^3^] the volume of the fin, and *β_n_* = (*n* − ½)π.

As explained in the derivation of this analytical solution, the summation series term decays faster than the other terms. Hence, when the system has transitioned from initial heating to heat propagation, it is appropriate to express the temperature as follows:

(2) $$T\left(x,\;t\right)-T_{e}=\frac{q_{0}L}{k}\frac{\left(e^{-m\left(2L-x\right)}+e^{-mx}\right)}{mL\left(1-e^{-2mL}\right)}-\frac{q_{0}L}{k}\frac{e^{-m^{2}\alpha t}}{m^{2}L^{2}}.$$

This expression can then be further simplified by utilizing the following identities:

(3) $$T_{S.S.}\left(x\right)\equiv \frac{q_{0}L}{k}\frac{\left(e^{-m\left(2L-x\right)}+e^{-mx}\right)}{mL\left(1-e^{-2mL}\right)}+T_{e},$$

where *T_S.S._*(*x*) is the steady-state temperature in the fin as a function of *x*;

(4) $$\;\Delta T^{*}\left(x\right)\equiv \frac{q_{0}L}{km^{2}L^{2}},$$

where ΔT^*^(*x*) is the increase in temperature between the initial and steady-state conditions; and

(5) $$\Omega \equiv m^{2}\alpha ,$$

where Ω [T^−1^] represents a time constant.

By using these identities, Equation (2) was rewritten to express the temperature at each cursor point in a simple exponential form. The resulting expression is a modified version of Newton’s law of cooling, and it is as follows:

(6) $$T\left(x,t\right)=T_{S.S.}\left(x\right)-\Delta T^{*}\left(x\right)e^{-\Omega t}.$$

This resulting expression can thus be used after a sufficient time has elapsed to allow for the decay of the summation series term of Equation (1). Subsequently, a least squares regression can be performed on the temperature data to determine *T_S.S._*(*x*) and ΔT^*^(*x*) for each cursor location and Ω for each beam. It should be noted that the thermography measurements in the presented experiments were inherently limited in accuracy. Therefore, the results obtained using this approach represent general trends rather than absolute values.

In this study, the first 1000 s of each experimental run was excluded from the analysis to allow for the decay of the summation series term in Equation (1). This time duration was found to be suitable for all temperature profiles, and further extension did not significantly affect the value of *T_S.S._*(*x*). The initial estimates for *T_S.S._*(*x*) and ΔT^*^(*x*) for each cursor point and Ω for each beam were obtained by plotting the experimental temperature progression data at each cursor and performing a rough regression. Subsequently, the regression sum of squares and total sum of squares were calculated for each time at each cursor, and an initial *R*^2^ value was determined for each cursor point. The Generalized Reduced Gradient (GRG) Nonlinear Solver functionality of Excel was then utilized to minimize the sum of (1 − *R*^2^) values for each cursor by varying *T_S.S._*(*x*), ΔT^*^(*x*), and Ω values. The regression equations were manually verified to ensure that the result provided was appropriate and not a local minimum for (1 − *R*^2^). In this way, *T_S.S._*(*x*) values were obtained for steady-state modeling for each beam. After determining the values for *T_S.S._*(*x*) at each cursor location, the steady-state model was applied to the conceptual setup, considering the beam as a cooling fin with one side exposed to a heat source and its top surface exposed to a convective cooling environment. The analytical solution also relies on Fourier’s equation and applies the same boundary conditions of zero heat transfer from the end of the fin away from the heat source (*x* = *L*). Another boundary condition is that the temperature of the fin at its junction with the heat source (*x* = 0) is identical to that of the heat source. Using these boundary conditions, the following analytical solution for *T_S.S._*(*x*) can be applied [55,56]:

(7) $$\frac{T_{S.S.}\left(x\right)-T_{e}}{T_{S.S.}(x=0)-T_{e}}=\frac{cosh(\left(1-\frac{x}{L}\right)mL)}{cosh(mL)},$$

where the value of *T_S.S._*(*x*) was determined at each of the six cursor position for each beam based on the unsteady-state model; the value of *T_S.S._*(*x =* 0) was estimated by using the value obtained for the first cursor position (near the heat source); and the dimensionless distance *x/L* was defined as the ratio of the distance from the first cursor to the distance between the first and last cursors (i.e., *x/L* = 0 near the heat source and 1 near the edge of the beam exposed to the convective environment).

The steady-state temperature at each cursor position was modeled using Equation (7) by performing a least squares regression. Excel’s GRG Nonlinear Solver was utilized to minimize (1 − *R*^2^) for each beam by varying *mL* and *T_e_*. The conceptual fin model used in this study assumed that the solid beams were exposed to a convective medium, with *T_e_* being set as the bulk temperature of the testing room. However, due to the complex structural features of the beams and the predominantly stagnant airflow in the surrounding environment, the formation of a complex boundary layer around the beams, along with associated convective and radiative effects, was anticipated. To address this, *T_e_* was treated as a parameter that needed to be fitted during the analysis. This simplification was found to produce consistent results in accounting for the complex interactions between the beam and its surrounding environment.

The analytical solution was made dimensionless by using the dimensionless temperature, $\theta \equiv \frac{T_{S.S.}\left(x\right)-T_{e}}{T_{S.S.}(x=0)-T_{e}}$, and the dimensionless length, x/L, yielding the following final form:

(8) $$\theta \left(\frac{x}{L}\right)=\frac{\cosh\left(\left(1-\frac{x}{L}\right)mL\right)}{\cosh\left(mL\right)}.$$

For each beam, the regression procedure yielded a fitted value for *mL*. Because it was anticipated that changes in internal architectures and cement ink type of the beams would primarily impact conductivity by modifying the pathways and cross-sectional area for heat flow, rather than surface-controlled thermal convection, it was assumed that the convection coefficient remained relatively constant across all beams. As a result, *mL* was considered inversely proportional to the square root of the conductive heat transfer coefficient, as demonstrated by the following:

(9) $$m\equiv \sqrt{\frac{hA_{h}}{kV}}.$$

To compare the impact of architecture and ink design on thermal conductivity, a dimensionless term called *k_app_* was introduced, defined as:

(10) $$k_{app}\equiv {\left(\frac{1}{mL}\right)}^{2}$$

and directly proportional to the effective conductive heat transfer coefficient. *k_app_* can then be directly compared between beams to determine the comparative effect of internal architecture and ink design on thermal conductivity.

A comprehensive discussion on the robustness of the results provided by this methodology and a statistical analysis using Welch’s *t*-test can be found in [50].

## 3. Results and Discussion

### 3.1. Thermal Imaging and Temperature Evolution

**Figure 4:**
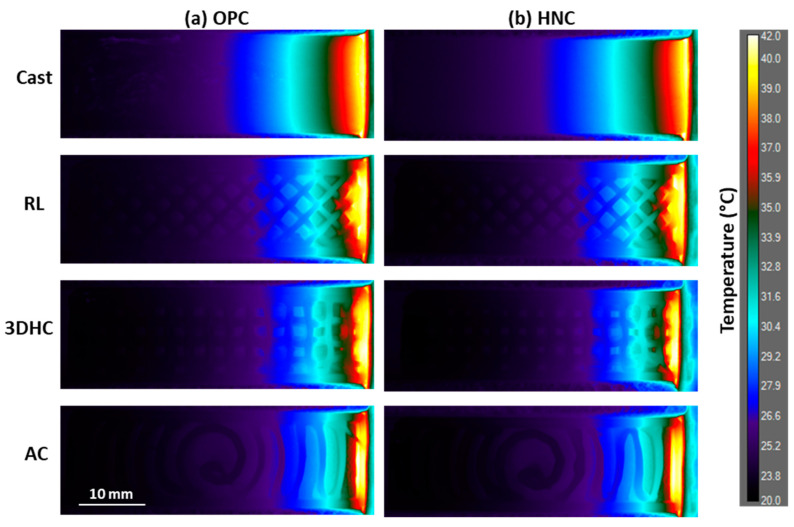
Infrared thermography images of (**a**) OPC ink beams and (**b**) HNC ink beams taken after 2000 s of contact with the heated source.

The influence of internal architecture design on the thermal response was overall greater than the influence of HNC incorporation in the cement ink. Infrared images of the top of the beams (Figure 4) revealed a distinct hierarchy among the different internal architectures, highlighting their significant influence on the thermal distribution. The beams exhibited varying degrees of heat penetration with the greatest heat penetration observed in the cast (solid) beams, followed by the RL beams, then the 3DHC beams, and finally the AC beams. However, the addition of HNC to the cement ink did not result in observable visual effect, and further examination of the temperature profiles was necessary.

**Figure 5:**
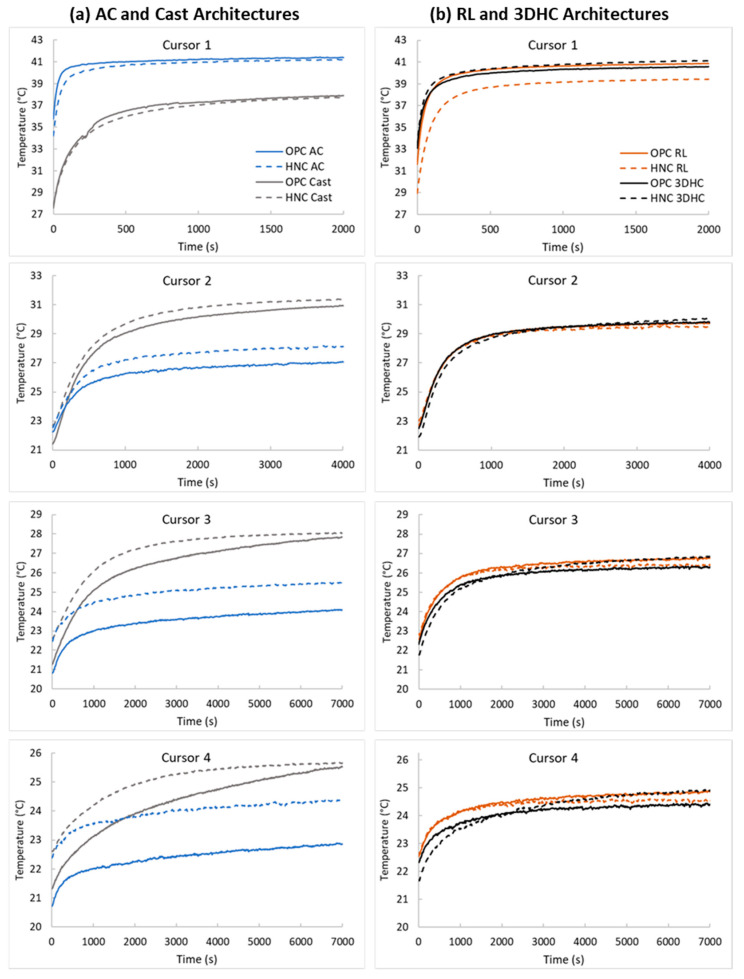
Representative temperature evolution as a function of time at Cursors 1–4, located at approximately 1 mm, 19 mm, 37 mm, and 61 mm from the heat source, respectively, for each internal architecture and cement ink type. (**a**) Archimedean chords (AC) and cast architectures. (**b**) Rectilinear (RL and three-dimensional honeycomb (3DHC) architectures. Note: the axis scales vary by cursor to emphasize the effects at different temperature ranges and timescales.

The AC architecture exhibited the most significant heat accumulation near the heat source (Cursor 1; Figure 5). This can be attributed to the absence of a direct heat conduction pathway between the filaments. The air cavity structures that run across the width of the beams acted as a thermal barrier, limiting the heat flow through the beams and creating a pathway for heat to be trapped. The AC beams also exhibited the most rapid initial rises in temperature at Cursor 1, followed by a rapid settling into a stable temperature profile. Conversely, the cast beams had the lowest degree of heat buildup at Cursor 1 because of the direct path for heat propagation uninterrupted by air cavities. As a result, the cast beams exhibited a more gradual initial temperature increase that slowly transitioned into a stable profile as the heat flux was able to continue to propagate without the higher buildup developed by the less direct flow path of the printed structures. However, this dynamic was reversed by Cursor 2 (approximately 19 mm away from the heat source) as the temperature profiles of the AC beams stabilized for each subsequent cursor at a lower temperature than that of the cast beams. This was due to the lower heat flux reaching that point in the AC beams. Additionally, in both the cast and AC beams, the use of the HNC ink led to higher temperature profiles compared to the OPC ink. This resulted from the ability of HNC to absorb heat, effectively increasing the heat capacity of the cement ink and raising the temperature. The effect of HNC incorporation on the temperature profile at Cursor 2 and beyond was more pronounced in the AC beams than in the cast beams. The use of HNC ink reduced the dissipation of heat in the voids formed between the spiral-shaped filaments of the AC architecture, resulting in a higher temperature increase in the AC beams compared to the cast beams. At all cursors, the temperature profiles for the RL and 3DHC beams were intermediate between those of the AC and cast beams. However, the relationship between the RL and 3DHC beams was found to be more intricate and was impacted by the cement ink used.

For the OPC ink, the temperature profiles at Cursors 1 and 2 were almost identical in both the RL and 3DHC architectures. However, as the distance from the heat source increased along the beams, the RL beams exhibited temperatures at subsequent cursors that were up to 0.5 °C higher than the 3DHC beams, suggesting a divergence in the thermal behavior between the two architectures. These results indicate that the RL beams made with the OPC ink exhibited a faster rate of heat transfer along their length compared to the 3DHC beams. The RL beams consisted of a grid-like pattern composed of intersecting filaments diagonally oriented at a 45-degree angle relative to the beam’s length. This arrangement increased the path for the heat to travel and created interconnected channels throughout the beam. These channels served as conduits for heat to flow through, allowing heat to travel not only within the solid portion of the beam but also through the channels. As a result, the overall heat conduction of the beam was enhanced. In contrast, the 3DHC beams consisted of a repeating cell structure that created a three-dimensional lattice and voids or air cavities that are surrounded by solid filaments. These voids interrupted the flow of heat through the beams and created a pathway for heat to be trapped or absorbed, resulting in a delayed release of heat and limiting the heat that could flow through the beams. As a result, the 3DHC beams reached lower temperatures away from the heat source than the RL beams when using the OPC ink.

For the HNC ink, the opposite trend in temperature profile was observed between the RL and 3DHC architectures. The 3DHC beams experienced a more rapid heat buildup at Cursor 1 and reached temperatures that were up to 0.5 °C higher than the RL beams at subsequent cursors. Furthermore, in the case of the 3DHC architecture with the HNC ink, the temperature at each cursor was higher compared to when the OPC ink was used. Conversely, the RL architecture showed the opposite trend, with a lower temperature at each cursor when the HNC ink was used instead of the OPC ink. The addition of HNC to the cement ink had distinct impacts on the behavior of the two architectures. In the RL beams, the insulating properties of HNC reduced the heat propagation through conduction, while the interconnected channels facilitated better heat dissipation. These channels provided pathways for the removal of heat from the beams, allowing for effective heat transfer with the surrounding environment. As a result, the combination of the insulating properties of HNC and the enhanced heat dissipation of the RL architecture contributed to lower overall temperatures compared to the OPC ink without HNC. In the 3DHC beams, the addition of HNC resulted in the accumulation of heat in the layers of filaments oriented across the width of the beams (i.e., perpendicular to the heat flow direction). This heat accumulation was facilitated by the heat absorption capacity of HNC, which increased the overall heat capacity of the cement ink. The 3DHC beams also had layers of filaments running along the length of the beam, creating a relatively direct heat conduction pathway in those layers. As a result, the combination of heat accumulation in the cross-filaments and the thermal energy storage provided by the air cavities led to temperatures that were higher at each cursor compared to when the OPC ink was used.

### 3.2. Temperature Profiles from Analytical Modeling

**Figure 6:**
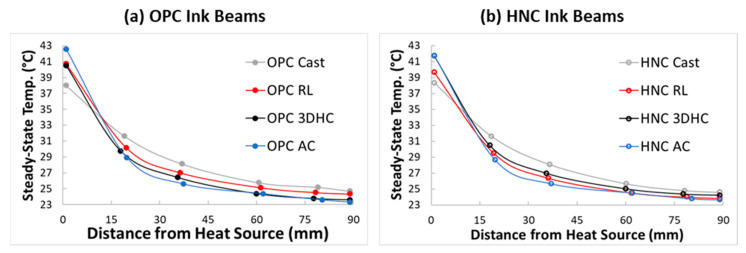
Steady-state temperature profiles for the (**a**) OPC ink beams and (**b**) HNC ink beams obtained from the analytical solution of the transient heat transfer model in a fin.

The analytical solution for transient heat transfer in a fin was employed to model the temperature change over time at each cursor location along the beams, extending beyond the first 1000 s. This enabled the determination of the steady-state temperature profiles along the length of the beams. Figure 6 presents the comparison of steady-state temperature profiles for the beams prepared with the OPC ink and HNC ink, as obtained from the unsteady-state analytical model. There was a noticeable difference in the steady-state temperatures along the length of the beams between the cast (solid) beams and 3D-printed beams. At Cursor 1, the cast beams had steady-state temperatures several degrees below those of the 3D-printed beams, indicating that less heat was trapped near the heating element in the absence of air voids. Beyond Cursor 1, the 3D-printed beams exhibited lower steady-state temperatures. The presence of voids and their degree of connectivity within the beam influenced the heat transfer process by creating pathways for air to be comparatively trapped (3DHC beams) or circulated (RL beams) within the structure. These findings align with previous research [57], which shows that the geometric structure of the air voids is a critical factor in regulating heat transfer. For the OPC ink, the RL beams exhibited overall higher steady-state temperatures compared to the 3DHC beams (Figure 6a). This behavior can be attributed to the more continuous and less obstructed pathways of the RL architecture, which facilitated more efficient heat transfer throughout the beam. In contrast, the 3DHC architecture, with its interconnected cells and voids, restricted the heat flow, leading to lower temperatures.

However, for the HNC ink, the opposite trend was observed, with the 3DHC beams showing overall higher steady-state temperatures compared to the RL beams (Figure 6b). The addition of HNC to the cement ink altered its thermal properties by increasing its overall heat capacity, allowing it to store more thermal energy. While the heat capacity of OPC powder is approximately 0.75 J/g·K [58], the higher heat capacity of HNC (around 0.9 J/g·K [59]), combined with the HNC’s tortuous nanostructure and the densification of the paste microstructure from its pozzolanic activity, increased the ability of the composite ink to retain heat. This increase in heat capacity led to a noticeable shift in the thermal behavior of the internal architectures. The design of the 3DHC architecture, characterized by layers of filaments oriented primarily perpendicular to the direction of the flow and the presence of isolated air cavities, effectively facilitated the accumulation and retention of heat within the beams. This behavior is consistent with the literature [60], where honeycomb structures are described as brick-like formations that insulate and absorb heat effectively. The increased heat capacity of the HNC ink further contributed to this effect by allowing the material to store more thermal energy, leading to higher steady-state temperatures. In contrast, the higher tortuosity and continuous heat pathways in the RL architecture allowed greater heat conduction and heat dissipation, resulting in lower steady-state temperatures in the RL beams compared to the 3DHC beams with HNC ink. This change in behavior may be evidence of a reduction in thermal transmittance with the addition of HNC, allowing more heat to be stored in the filaments oriented along the width of the beam in the 3DHC architecture, while less heat is conducted through the tortuous pathway of the RL architecture filaments compared to the OPC ink. These results underscore that the interaction between the material composition and internal architectures plays an important role in determining thermal performance.

### 3.3. Apparent Thermal Conductivity

The steady-state temperatures, derived from the analytical solution of transient heat transfer in a fin, were employed alongside the analytical solution for steady-state heat transfer in the same conceptual fin model to determine the apparent thermal conductivity of each beam. The resulting apparent thermal conductivity values, as defined in Equation (10), are summarized in Table 2. There was a clear hierarchy of apparent thermal conductivity among the different internal architectures. This hierarchy was primarily influenced by the spatial configuration and interconnectivity of the voids within the beams. The cast (solid) beams exhibited the highest apparent thermal conductivity, followed by the RL, 3DHC, and AC beams in descending order. The differences in apparent thermal conductivity between the different internal architectures were statistically significant at a 95% confidence level, as determined by Welch’s *t*-test with a significance level of α = 0.05. For both cement inks, the AC architecture led to the most significant decrease in apparent thermal conductivity, with a reduction by a factor of 4 relative to the cast beams. The lattice structure created by the chords hindered the movement of heat and decreased the overall thermal conductivity of the beams. The RL architecture exhibited a 10–15% higher apparent thermal conductivity than the 3DHC architecture and a 35–40% higher apparent thermal conductivity than the AC architecture. The RL architecture, with its interconnected channels between filaments, facilitated heat transfer within the beams, leading in increased apparent thermal conductivity compared to the AC and 3DHC architectures. These findings are consistent with the literature [61], which indicates that the structure of the RL architecture leads to increased thermal conductivity compared to honeycomb-type architectures. The presence of isolated air cavities in the 3DHC architecture, which acted as insulators and thermal energy storage, reduced the heat flow, leading to a decrease in the apparent thermal conductivity of the beams.

In comparison to the internal architectures, the addition of HNC to the cement ink caused a relatively smaller reduction in the apparent thermal conductivity. The extent of this reduction varied depending on the architecture. The AC beams showed the greatest reduction (14%), followed by the cast (solid) beams (8%), and the 3DHC beams (5%). This reduction in thermal conductivity aligned with the insulating and heat-absorbing properties of HNC, as well as the previous literature, which has shown that incorporating nanoclay into cementitious materials can lead to a decrease in thermal conductivity [46,47]. Interestingly, the RL beams exhibited a distinct behavior, showing no statistically significant differences in the apparent thermal conductivity compared to the OPC ink when HNC was incorporated. The enhanced heat dissipation and structural characteristics of the RL architecture had a greater influence on heat transfer compared to the insulating properties of HNC, resulting in altered conduction through the filaments and increased heat dissipation. In contrast, for the AC and 3DHC architectures, the presence of HNC further amplified the influence of their structural characteristics on heat transfer. The diverse effects of incorporating HNC into cement ink across the different architectural designs underscore the significance of the internal architecture design and cement ink properties in governing heat transfer within the 3D-printed structure. When evaluating the feasibility of integrating HNC into cement ink, consideration should be given to the specific internal architecture and intended application.

## 4. Conclusions

The impact of internal architecture and ink formulation on the thermal properties of 3D-printed cement composite beams was evaluated using thermal experiments, infrared thermal imaging, and a one-dimensional heat transfer model to determine apparent thermal conductivity. The following conclusions were drawn:

- The internal architectures had a greater impact on thermal behavior than HNC, reducing apparent thermal conductivity by up to 75%, while HNC incorporation reduced it by up to 14%.
- The following clear progression was observed for internal architectures in order of decreasing apparent thermal conductivity, regardless of the cement ink used: cast, RL, 3DHC, and AC, with air voids and fewer direct conduction pathways resulting in reduced conductivity.
- Distinct thermal behaviors were observed among the internal architectures: the RL architecture facilitated heat transfer, exhibiting 10–15% higher apparent thermal conductivity than the 3DHC architecture and a 35–40% higher apparent thermal conductivity than the AC architecture. The 3DHC architecture retained heat, while the unbridged air voids in the AC architecture disrupted heat flow.

The study shows that internal architecture and cement ink formulation can control heat transfer and thermal conductivity of cement-based elements. The proposed experimental setup, combined with a heat transfer model, is effective for analyzing various internal architectures and material compositions. This methodology can be scaled to enhance the thermal performance of 3D-printed structures at construction-relevant scales. Further research is needed to examine the connection between internal architectures, hollow cavities, and reinforcing materials.

## Figures and Tables

**Table 1:** Formulations with 100 g of solids as the basis for the OPC and HNC inks.

Ink Type	OPC	HNC	Water	VMA 362	Glenium 7700	UW 450
OPC Ink	100 g	-	30 g	0.90 g	0.35 g	1.00 g
HNC Ink	95 g	5 g	30 g	0.90 g	0.35 g	1.00 g

**Table 2:** Average dimensionless apparent thermal conductivity, *k_app_*, values with standard deviations for each beam type (based on three replicates).

	Architecture Type			
Ink Type	Cast	RL	3DHC	AC
OPC	0.1362 ± 0.0081	0.0456 ± 0.0020	0.0408 ± 0.0013	0.0329 ± 0.0013
HNC	0.1251 ± 0.0070	0.0443 ± 0.0018	0.0385 ± 0.0005	0.0282 ± 0.0020

## Data Availability

The data presented in this study are available on request from the corresponding author due to ongoing study.

## References

[1] IEA (2018). The Future of Cooling: Opportunities for Energy-Efficient Air Conditioning.

[2] Chung S.-Y., Abd Elrahman M., Stephan D. (2016). Investigation of the effects of anisotropic pores on material properties of insulating concrete using computed tomography and probabilistic methods. Energy Build..

[3] Peruzzi A.d.P., Rossignolo J.A., Kahn H. (2018). Air-entrained concrete: Relationship between thermal conductivity and pore distribution analyzed by X-ray tomography. J. Civ. Eng. Archit..

[4] Kim H.-K., Jeon J., Lee H.-K. (2012). Workability, and mechanical, acoustic and thermal properties of lightweight aggregate concrete with a high volume of entrained air. Constr. Build. Mater..

[5] Hossain M.A. (2020). Mechanical and thermal performance of cement mortar incorporating super absorbent polymer (SAP). Civ. Eng. J..

[6] Jeong Y.-W., Koh T.-H., Youm K.-S., Moon J. (2017). Experimental evaluation of thermal performance and durability of thermally-enhanced concretes. Appl. Sci..

[7] Samson G., Phelipot-Mardelé A., Lanos C. (2017). A review of thermomechanical properties of lightweight concrete. Mag. Concr. Res..

[8] Zeng Q., Fang R., Li H., Peng Y., Wang J. (2019). Tailoring the thermal and mechanical properties of lightweight cement-based composites by macro and micro fillers. Cem. Concr. Compos..

[9] Zeng Q., Mao T., Li H., Peng Y. (2018). Thermally insulating lightweight cement-based composites incorporating glass beads and nano-silica aerogels for sustainably energy-saving buildings. Energy Build..

[10] Thompson B.R., Horozov T.S., Stoyanov S.D., Paunov V.N. (2018). Hierarchically porous composites fabricated by hydrogel templating and viscous trapping techniques. Mater. Des..

[11] Xiao P., Yifeng Z., Peng W., Dan L. (2019). Estimation of thermal conduction in hollow-glass-beads-filled cement-based composites by variational asymptotic homogenization method. Appl. Therm. Eng..

[12] Lu J., Jiang J., Lu Z., Li J., Niu Y., Yang Y. (2020). Pore structure and hardened properties of aerogel/cement composites based on nanosilica and surface modification. Constr. Build. Mater..

[13] Záleská M., Pokorný J., Pavlíková M., Pavlík Z. (2017). Thermal properties of light-weight concrete with waste polypropylene aggregate. AIP Conf. Proc..

[14] Xie Y., Li J., Lu Z., Jiang J., Niu Y. (2018). Effects of bentonite slurry on air-void structure and properties of foamed concrete. Constr. Build. Mater..

[15] Zhao Z., Qu X., Li F., Wei J. (2018). Effects of steel slag and silica fume additions on compressive strength and thermal properties of lime-fly ash pastes. Constr. Build. Mater..

[16] Ali K., Qureshi M.I., Saleem S., Khan S.U. (2021). Effect of waste electronic plastic and silica fume on mechanical properties and thermal performance of concrete. Constr. Build. Mater..

[17] Bentz D.P., Peltz M.A., Duran-Herrera A., Valdez P., Juarez C. (2011). Thermal properties of high-volume fly ash mortars and concretes. J. Build. Phys..

[18] Vásquez-Molina D., Mejía-Arcila J.M., Gutiérrez R.M.-d. (2016). Mechanical and thermal performance of a geopolymeric and hybrid material based on fly ash. Dyna.

[19] Sharma R., Jang J.-G., Hu J.-W. (2022). Phase-change materials in concrete: Opportunities and challenges for sustainable construction and building materials. Materials.

[20] Korniejenko K., Nykiel M., Choinska M., Jexembayeva A., Konkanov M., Aruova L. (2024). An Overview of Phase Change Materials and Their Applications in Pavement. Energies.

[21] Marais H., Christen H., Cho S., De Villiers W., Van Zijl G. (2021). Computational assessment of thermal performance of 3D printed concrete wall structures with cavities. J. Build. Eng..

[22] Ayegba B.O., Egbe K.-J.I., Matin Nazar A., Huang M., Hariri-Ardebili M.A. (2022). Resource Efficiency and Thermal Comfort of 3D Printable Concrete Building Envelopes Optimized by Performance Enhancing Insulation: A Numerical Study. Energies.

[23] Suntharalingam T., Gatheeshgar P., Upasiri I., Poologanathan K., Nagaratnam B., Rajanayagam H., Navaratnam S. (2021). Numerical Study of Fire and Energy Performance of Innovative Light-Weight 3D Printed Concrete Wall Configurations in Modular Building System. Sustainability.

[24] Suntharalingam T., Upasiri I., Nagaratnam B., Poologanathan K., Gatheeshgar P., Tsavdaridis K.D., Nuwanthika D. (2022). Finite Element Modelling to Predict the Fire Performance of Bio-Inspired 3D-Printed Concrete Wall Panels Exposed to Realistic Fire. Buildings.

[25] Suntharalingam T., Upasiri I., Gatheeshgar P., Poologanathan K., Nagaratnam B., Santos P., Rajanayagam H. (2021). Energy Performance of 3D-Printed Concrete Walls: A Numerical Study. Buildings.

[26] Yang S., Wi S., Park J.H., Cho H.M., Kim S. (2019). Novel proposal to overcome insulation limitations due to nonlinear structures using 3D printing: Hybrid heat-storage system. Energy Build..

[27] Gosselin C., Duballet R., Roux P., Gaudillière N., Dirrenberger J., Morel P. (2016). Large-scale 3D printing of ultra-high performance concrete–a new processing route for architects and builders. Mater. Des..

[28] Alghamdi H., Neithalath N. (2019). Synthesis and characterization of 3D-printable geopolymeric foams for thermally efficient building envelope materials. Cem. Concr. Compos..

[29] Ma G., Ruhan A., Xie P., Pan Z., Wang L., Hower J.C. (2022). 3D-printable aerogel-incorporated concrete: Anisotropy influence on physical, mechanical, and thermal insulation properties. Constr. Build. Mater..

[30] de Rubeis T. (2022). 3D-Printed Blocks: Thermal Performance Analysis and Opportunities for Insulating Materials. Sustainability.

[31] Sovetova M., Calautit J.K. (2024). Thermal and energy efficiency in 3D-printed Buildings: Review of geometric Design, materials and printing processes. Energy Build..

[32] Ramakrishnan S., Muthukrishnan S., Sanjayan J., Pasupathy K. (2021). Concrete 3D printing of lightweight elements using hollow-core extrusion of filaments. Cem. Concr. Compos..

[33] Kawashima S., Wang K., Ferron R.D., Kim J.H., Tregger N., Shah S. (2021). A review of the effect of nanoclays on the fresh and hardened properties of cement-based materials. Cem. Concr. Res..

[34] Qian Y., Ma S., Kawashima S., De Schutter G. (2019). Rheological characterization of the viscoelastic solid-like properties of fresh cement pastes with nanoclay addition. Theor. Appl. Fract. Mech..

[35] Kaushik S., Sonebi M., Amato G., Perrot A., Das U.K. (2022). Influence of nanoclay on the fresh and rheological behaviour of 3D printing mortar. Mater. Today Proc..

[36] Pan T., Jiang Y., He H., Wang Y., Yin K. (2021). Effect of Structural Build-Up on Interlayer Bond Strength of 3D Printed Cement Mortars. Materials.

[37] Natanzi A.S., McNally C. (2020). Characterising Concrete Mixes for 3D Printing. Second RILEM International Conference on Concrete and Digital Fabrication, Proceedings of the Digital Concrete 2020, Eindhoven, The Netherlands, 6–8 July 2020.

[38] Panda B., Unluer C., Tan M.J. (2019). Extrusion and rheology characterization of geopolymer nanocomposites used in 3D printing. Compos. Part B Eng..

[39] Sonebi M., Dedenis M., Amziane S., Abdalqader A., Perrot A. (2021). Effect of Red Mud, Nanoclay, and Natural Fiber on Fresh and Rheological Properties of Three-Dimensional Concrete Printing. ACI Mater. J..

[40] Zhang Y., Zhang Y., Liu G., Yang Y., Wu M., Pang B. (2018). Fresh properties of a novel 3D printing concrete ink. Constr. Build. Mater..

[41] Sajadi S.M., Boul P.J., Thaemlitz C., Meiyazhagan A.K., Puthirath A.B., Tiwary C.S., Rahman M.M., Ajayan P.M. (2019). Direct ink writing of cement structures modified with nanoscale Additive. Adv. Eng. Mater..

[42] Hakamy A., Shaikh F., Low I.M. (2015). Characteristics of nanoclay and calcined nanoclay-cement nanocomposites. Compos. Part B Eng..

[43] Farzadnia N., Ali A.A.A., Demirboga R., Anwar M.P. (2013). Effect of halloysite nanoclay on mechanical properties, thermal behavior and microstructure of cement mortars. Cem. Concr. Res..

[44] Allalou S., Kheribet R., Benmounah A. (2019). Effects of calcined halloysite nano-clay on the mechanical properties and microstructure of low-clinker cement mortar. Case Stud. Constr. Mater..

[45] Wang W.-C. (2017). Compressive strength and thermal conductivity of concrete with nanoclay under Various High-Temperatures. Constr. Build. Mater..

[46] Cho J., Waetzig G.R., Udayakantha M., Hong C.Y., Banerjee S. (2018). Incorporation of hydroxyethylcellulose-functionalized halloysite as a means of decreasing the thermal conductivity of oilwell cement. Sci. Rep..

[47] Udayakantha M., Cho J., Liu K.-W., Mukhopadhyay A., Gupta S., Hong C.Y., Banerjee S. (2019). An evaluation of the reduction of heat loss enabled by halloysite modification of oilwell cement. Eng. Res. Express.

[48] Haw T.T., Hart F., Rashidi A., Pasbakhsh P. (2020). Sustainable cementitious composites reinforced with metakaolin and halloysite nanotubes for construction and building applications. Appl. Clay Sci..

[49] Liu H., Jin J., Yu Y., Liu H., Liu S., Shen J., Xia X., Ji H. (2020). Influence of halloysite nanotube on hydration products and mechanical properties of oil well cement slurries with nano-silica. Constr. Build. Mater..

[50] Kosson M.T. (2022). Chemo-Mechanical and Thermal Behavior of 3D Printed Cement Composites. Ph.D. Thesis.

[51] BASF (2018). MasterGlenium 7700.

[52] BASF (2018). MasterMatrix UW 450.

[53] BASF (2019). MasterMatrix VMA 362.

[54] Singh T., Shrivastava S., Ber H.S. (2013). Analysis of unsteady heat conduction through short fin with applicability of quasi theory. Int. J. Mech. Eng. Robot. Res..

[55] Spakovszky Z.S. 18.2 Heat Transfer From a Fin. https://web.mit.edu/16.unified/www/FALL/thermodynamics/notes/node128.html.

[56] Lienhard J.H., Lienhard J.H. (2024). A Heat Transfer Textbook.

[57] Anwajler B., Szołomicki J., Noszczyk P., Baryś M. (2024). The Potential of 3D Printing in Thermal Insulating Composite Materials—Experimental Determination of the Impact of the Geometry on Thermal Resistance. Materials.

[58] Bentz D.P. (2007). Transient plane source measurements of the thermal properties of hydrating cement pastes. Mater. Struct..

[59] DeArmitt C. Halloysite Clay Nanotubes: Introduction to Halloysite Filler. https://phantomplastics.com/functional-fillers/halloysite-filler-gives-unique-properties-to-plastics-and-coatings/.

[60] Zhang T., Zhang X., Zhang S., Wang L., Yu D., Wang W. (2024). 3D Printing-Assisted Honeycomb-Structured Bricklike Aerogels Applied for Infrared Stealth with Good Thermal Management. ACS Appl. Polym. Mater..

[61] Güngör Ş. (2023). Experimental investigations on the thermal performance of additively manufactured porous topologies. Dokuz Eylül Üniversitesi Mühendislik Fakültesi Fen Ve Mühendislik Derg..

